# Changes in Pelvic Floor Ultrasonographic Features after Flat Magnetic Stimulation in Women with Chronic Pelvic Pain and Levator Ani Muscle Hypertonicity

**DOI:** 10.3390/medicina60030374

**Published:** 2024-02-23

**Authors:** Marta Barba, Alice Cola, Desirèe De Vicari, Clarissa Costa, Giorgio La Greca, Annalisa Vigna, Silvia Volontè, Matteo Frigerio, Stefano Terzoni, Serena Maruccia

**Affiliations:** 1 Department of Gynecology, IRCCS San Gerardo dei Tintori, University of Milano-Bicocca, 20900 Monza, Italy; m.barba8792@gmail.com (M.B.); alice.cola1@gmail.com (A.C.); d.devicari@campus.unimib.it (D.D.V.); c.costa14@campus.unimib.it (C.C.); s.volonte6@campus.unimib.it (S.V.); 2 Department of Coloproctology and Pelvic Floor Surgery, IRCCS Policlinico San Donato Hospital, 20097 Milan, Italy; giorgiolagreca@gmail.com; 3 Department of Gynecology, IRCCS Policlinico San Martino, University of Genova, 16132 Genova, Italy; annalisa.vigna.92@gmail.com; 4 Department of Biomedical Health Sciences, University of Milan, 20133 Milan, Italy; stefano.terzoni@unimi.it; 5 Department of Urology, ASST Santi Paolo e Carlo, San Paolo Hospital, 20142 Milan, Italy; serena.maruccia@gmail.com

**Keywords:** flat magnetic stimulation, chronic pelvic pain, vulvodynia, pelvic floor hypertonicity, ultrasound

## Abstract

*Background and Objectives:* Chronic pelvic pain (CPP) represents a major public health problem for women with a significant impact on their quality of life. In many cases of CPP, due to gynecological causes—such as endometriosis and vulvodynia—improper pelvic floor muscle relaxation can be identified. Treatment of CPP with pelvic floor hypertonicity (PFH) usually involves a multimodal approach. Traditional magnetic stimulation has been proposed as medical technology to manage muscle hypertonicity and pelvic pain conditions through nerve stimulation, neuromodulation, and muscle relaxation. New Flat Magnetic Stimulation (FMS)—which involves homogeneous rather than curved electromagnetic fields—has the potential to induce sacral S2–S4 roots neuromodulation, muscle decontraction, and blood circulation improvement. However, the benefits of this new technology on chronic pelvic pain symptoms and biometrical muscular parameters are poorly known. In this study, we want to evaluate the modification of the sonographic aspect of the levator ani muscle before and after treatment with Flat Magnetic Stimulation in women with chronic pelvic pain and levator ani hypertonicity, along with symptoms evolution. *Materials and Methods:* A prospective observational study was carried out in a tertiary-level Urogynaecology department and included women with CPP and PFH. Approval from the local Ethics Committee was obtained before the start of the study (protocol code: MAGCHAIR). At the baseline, the intensity of pelvic pain was measured using a 10 cm visual analog scale (VAS), and patients were asked to evaluate their pelvic floor symptoms severity by answering the question, “How much do your pelvic floor symptoms bother you?” on a 5-answer Likert scale. Transperineal ultrasound (TPU) was performed to assess anorectal angle (ARA) and levator ani muscle minimal plane distance (LAMD). Treatment involved Flat Magnetic Stimulation alone or with concomitant local or systemic pharmacological therapy, depending on the patient’s preferences. FMS was delivered with the DR ARNOLD system (DEKA M.E.L.A. Calenzano, Italy). After the treatment, patients were asked again to score the intensity of pelvic pain using the 10 cm visual analog scale (VAS) and to evaluate the severity of their pelvic floor symptoms on the 5-answer Likert scale. Patients underwent TPU to assess anorectal angle (ARA) and levator ani muscle minimal plane distance (LAMD). *Results:* In total, 11 patients completed baseline evaluation, treatment, and postoperative evaluation in the period of interest. All patients underwent eight sessions of Flat Magnetic Stimulation according to the protocol. Adjuvant pharmacological treatment was used in five (45.5%) patients. Specifically, we observed a significant increase in both ARA and LAMD comparing baseline and post-treatment measurements (*p* < 0.001). Quality of life scale scores at baseline and after treatment demonstrated a significant improvement in both tools (*p* < 0.0001). *Conclusions:* Flat Magnetic Stimulation, with or without adjuvant pharmacological treatment, demonstrated safety and efficacy in reducing pelvic floor hypertonicity, resulting in improvement in symptoms’ severity and sonographic parameters of muscular spasm.

## 1. Introduction

Chronic pelvic pain (CPP) represents a major public health problem for women with a significant impact on their quality of life [1]. It is defined as pain originating from the pelvis, typically with a duration of more than 6 months, and is often associated with urinary, sexual, and bowel symptoms or with gynecologic dysfunction, which can have negative cognitive, behavioral, sexual, and emotional consequences [2]. It is estimated to affect 26% of the world’s female population, with estimated costs in the USA of USD 5.8 billion in 2020.

CPP is a multifactorial disorder, and pain may originate from gynecological, gastrointestinal, pelvic, musculoskeletal, or nervous systems [3]. Chronic pelvic pain syndrome (CPPS) is a diagnosis of exclusion based on the presence of CPP in the absence of a confirmed infection or a local pathology accounting for the pain [4]. In the absence of well-defined pathology, CPPS is classified according to symptoms, signs, and, where possible, investigations. However, in many cases, chronic pain may continue even after the initial cause has been cured. Gynecological causes of chronic pelvic pain can be divided into two groups: disorders of the external genitals (e.g., vulvodynia, primary vestibular pain syndrome, primary clitoral pain syndrome) and internal pelvic pain syndromes (e.g., endometriosis-associated pain syndrome and primary dysmenorrhoea) [5]. Among all, the most common chronic gynecologic pain syndromes are endometriosis and vulvodynia. 

Interestingly, women with endometriosis and vulvodynia are more likely to have improper pelvic floor muscle relaxation [6]. Painful or chronic muscular overload can cause the growth of hyperirritable areas called myofascial trigger points (MTrPs) within the pelvic floor and adjacent (abdominal, gluteal, and iliopsoas) muscles. An active MTrP is clinically associated with spontaneous pain in the surrounding tissue and/or to distant sites in specific referred pain patterns [7]. Pain is aggravated by trigger point pressure or sustained/repeated pelvic floor muscle contraction, such as pain related to voiding, defecation, or sexual intercourse. [4]. This condition has also been defined as pelvic floor hypertonicity (PFH). Several terms are used for PFH in the literature, such as pelvic floor spasm, nonrelaxing pelvic floor, and overactivity. Currently, the International Urogynecological Association (IUGA)/International Continence Society (ICS) defines the term “non-neurogenic hypertonicity” as an increase in muscle tone related to the contractile or viscoelastic components that can be associated with either elevated contractile activity and/or passive stiffness in the muscle.

PFH can be primary or secondary to peripheral and central sensitization resulting from other pain conditions, such as acute or chronic injury to one or more musculoskeletal components in the pelvic floor and surrounding structures. Pelvic surgery, traumatic vaginal delivery, traumatic injury of the back or pelvis, gait disturbances, pelvic pain, experienced threat, and (chronic) stress are found to be associated with PFH [8,9,10]. PFH is also assumed to be related to wrong behaviors, for example, voluntary holding to inhibit micturition or defecation or to avoid incontinence. This might be related to habit, lifestyle, and/or stressful occupation. Laan et al. conceptualized PFH as a symptom of chronic activation of the defensive stress system and should thus be regarded as a physical manifestation of emotional dysregulation [11].

Given the multifactorial nature of chronic pelvic pain, diagnosis should include a biopsychosocial approach [12]. The evaluation should start with a detailed history collection of the pain onset and progression, location, frequency, distribution, quality, the severity of all painful sites, coexisting pelvic and non-pelvic pain conditions [12,13]; a complete review of medical diagnoses, past surgeries, pain triggers (activity, menstruation, intercourse, and stress); and urological, gastroenterological, gynecologic, and myofascial symptoms [13]. Screening for bladder pain syndrome or interstitial cystitis and irritable bowel syndrome is specifically recommended by international guidelines [12,13].

Clinically, there is no consensus on diagnostic criteria for PFH. Vaginal examination is easy to perform and is considered the reference test to assess PFH [14,15]. Tenderness on examination should be considered an uncommon finding in asymptomatic individuals [16]. The vaginal examination represents the first level examination to be able to evaluate pelvic floor pathologies with good inter- and intra-rater reliability [17,18,19]. During the gynecological examination, muscle tone in response to pressure and/or voluntary contractility of the muscle and strength, resistance, repeatability, co-contraction, and relaxation capacity can be assessed [20,21]. There is no single accepted or standardized method to objectively assess muscle tone; furthermore, there are no normative values [20]. In some cases, instrumental tests such as surface electromyography (s-EMG) and dynamometry are associated with a gynecological examination to make a more objective assessment of the pelvic floor muscles [22,23].

A recent systematic review analyzed different clinical and instrumental diagnostic tools [24]. For example, a digital technique for pelvic floor muscle assessment—the PERFECT scheme—has been described. PERFECT is an acronym with P representing power (or pressure measured by a manometric perineometer), E = endurance, R = repetitions, F = fast contractions, and, finally, ECT = every contraction timed, which has demonstrated great reliability and validity as a pelvic floor assessment tool [25]. Dynamometry has been used to evaluate the endurance and strength of the pelvic floor muscles [26,27], but its use is still limited by the difficulty of accessing the device outside of a research context and by the limited experience of clinicians. Vaginal manometry is a second-level diagnostic tool that allows objective assessment of muscle pressure/resistance [28,29] compared to digital examination [29]. To make a subjective assessment of PFH, it is possible to use different types of questionnaires such as the Pelvic Floor Distress Inventory [30,31,32], the Pelvic Floor Impact Questionnaire [30,32], the Pelvic Pain, Urgency and Frequency [32], Central Sensitization Inventory [33], and the McGill Pain Questionnaire [34]. The modified Oxford scale, through a digital visit, [26,27] allows the muscular strength of the pelvic floor to be quantified as 0, no contraction; 1, flickering; 2, weak; 3, moderate; 4, good; and 5, strong [35]. Electromyography (EMG) has also been used to evaluate nerve transmission to the muscle [36,37,38]. The limitations to the use of this equipment are mainly the limited experience in its use and the lack of a suitable vaginal probe [38]. Both ultrasound (both transperineal and transvaginal) [39,40,41] and magnetic resonance imaging (MRI) [41] are emerging imaging modalities for evaluating pelvic floor muscle morphometry.

Both MRI and transvaginal/transperineal ultrasound allow the evaluation of pelvic floor disorders, but ultrasound is more accessible and easier to perform. In particular, pelvic floor ultrasound by transperineal route offers some advantages. Transperineal ultrasound (TPU) is a non-invasive, easy-to-use, and safe technique that dynamically evaluates the pelvic floor area. It is also a reproducible tool for assessing pelvic floor muscle integrity, contraction, and relaxation [42]. Transperineal sonographic equipment includes a B-mode compatible two-dimensional (2D) ultrasound system with a cine-loop function, a convex transducer with a frequency of 3.5–7.5 MHz, and a video transmission system. In order to perform a correct perineal ultrasound, a lithotomy position with the hips flexed and slightly abducted and the heels close to the buttocks with the lumbar spine in neutral may be preferable. The gel is applied directly to the probe, which is covered with a condom, a medical glove, or a probe cover. Only then will the examination be carried out. The midsagittal view of the pelvic cavity is obtained by positioning the probe orthogonally and vertically on the centrum of the perineum. The examination is usually painless, and there is no discomfort after placing the probe over the perineum and pubic symphysis. The standard mid-sagittal view shows, from left to right of the monitor, the pubic symphysis, urethra and bladder neck, vagina, and anorectal junction. Moreover, this scan allows direct and indirect evaluation of levator ani complex contractility status.

Different sonographic markers have been proposed to evaluate levator ani contractility status on 2D-transperineal ultrasound. The distance between the inferior border of the pubic symphysis to the medial border of the levator ani (puborectalis muscle) has been previously evaluated. In order to measure the levator ani muscle minimal plane distance (LAMD), a line from the inferior limit of the pubic symphysis to the anorectal junction should be drawn, representing a standardized antero-posterior dimension of the pelvic hiatus on 2D imaging. On the same scan, the anorectal angle can be estimated. The more the levator ani is contracted (such as during Valsalva or in case of hypertonicity), the more the anorectal angle is expected to be accentuated [42].

Once the diagnosis of pelvic floor hypertonicity is established, treatment usually requires a staged and multimodal approach and may comprehend pelvic floor rehabilitation (e.g., biofeedback, electrical stimulation, magnetic stimulation, pelvic muscle relaxation, and general relaxation training), first-line pharmacological treatment (e.g., Gabapentin, tricyclic antidepressants, muscle relaxant, vaginal estrogen, and NSAIDS); second-line pharmacological treatment (e.g., pregabalin, serotonin–norepinephrine reuptake inhibitors, and vaginal diazepam); third-line pharmacological treatment (e.g., opioids, topical anesthetic, cannabis); neuromodulation (sacral neuromodulation; S2–S4 roots magnetic modulation); local injections (local anesthetics/glucocorticoids or botulinum toxin).

Magnetic stimulation (MS) may successfully manage muscle hypertonicity conditions and related chronic pelvic pain. It generates an electrical field, resulting in nerve stimulation, neuromodulation, and muscle relaxation. Recently, magnetic stimulator technology witnessed big advancements, including Flat Magnetic Stimulation (FMS). In recent years, technological progress has led to improvements in scientific equipment. In particular, Flat Magnetic Stimulation (FMS) allows the generation of electromagnetic fields with a homogeneous profile, which is useful for the treatment of the pelvic area. The innovative feature of the FMS is the homogeneous distribution of the magnetic field. This homogeneous stimulation generates areas of uniform intensity, and, therefore, the muscle works with the same intensity in all fields. It also allows for greater recruitment of muscle fibers without creating unstimulated/recruited areas. This is believed to be associated with greater treatment efficacy compared to standard MS. This involves homogeneous, rather than curved, electromagnetic fields, which are able to standardize the effect on the entire pelvic area [43]. Due to the equal distribution and intensity of stimulation, FMS allows greater recruitment of muscle fibers without leaving areas of inconstant/suboptimal stimulation, leading to substantial advantages compared with standard magnetic stimulation treatment [44]. The interaction between the magnetic field and the neuromuscular tissue induces electrical currents, which may induce sacral S2–S4 roots neuromodulation, muscle decontraction, and blood circulation improvement. However, the benefits of this new technology on chronic pelvic pain related to pelvic floor hypertonicity are poorly known. 

In this study, we want to evaluate the modification of the sonographic aspect of the levator ani muscle before and after treatment with Flat Magnetic Stimulation in women with chronic pelvic pain and levator ani hypertonicity, along with symptoms evolution.

## 2. Materials and Methods

A prospective observational study was carried out in a tertiary-level Urogynaecology department and included women with CPPS. Approval from the local Ethics Committee was obtained before the start of the study (protocol code: MAGCHAIR). Recruitment occurred from September 2023 to November 2023 in the gynecologic outpatients in Fondazione IRCCS San Gerardo dei Tintori, Monza, Italy. 

During the study period, patients with chronic pelvic pain underwent a clinical interview to investigate the concomitant presence of lower urinary tract symptoms, bowel symptoms, or sexual dysfunction. All definitions conformed to IUGA/ICS terminology [20]. A gynecological evaluation was performed, and hypertonicity of the pelvic floor muscles was described. Patients who were younger than 18 years of age, pregnant, had congestive heart failure, arrhythmia, a history of malignancy, recent deep vein thrombosis, fever, acute inflammatory disease, or fractures in the treatment area were excluded from the study. Furthermore, as previously stated, women with insufficient knowledge of the Italian language, weighing more than 160 kg, or with neurostimulators, pacemakers, defibrillators, or ferromagnetic prostheses were excluded.

At baseline, pelvic pain intensity was assessed using a 10 cm visual analog scale (VAS), where the left end of the scale (score = 0) indicated “no symptoms” and the right end indicated “very severe symptoms” it could be (score = 100) [45]. Additionally, patients were asked to rate the intensity of their pelvic floor symptoms by answering the question “How much do your pelvic floor symptoms bother you?” on a 5-response Likert scale with the following choice of answers: “1, not at all”; “2, a little”; “3, moderate”; “4, a lot”; and “5, very much” [46].

**Figure 1:**
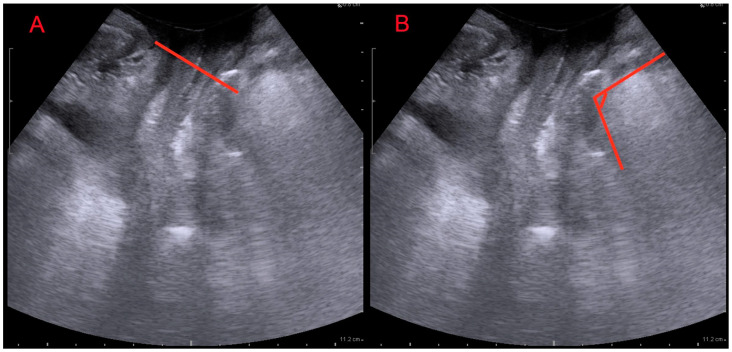
Translabial ultrasound: midsagittal view. (**A**): levator ani muscle minimal plane distance (LAMD) defined as the minimal distance between the hyperechogenic posterior aspect of the symphysis pubis and the hyperechogenic anterior border of the levator ani muscle just posterior to the anorectal angle; (**B**): anorectal angle (ARA) measured as the angle between the posterior edge of the rectum and the posterior edge of the anal canal.

From an instrumental point of view, patients underwent TPU to assess anorectal angle (ARA) and levator ani muscle minimal plane distance (LAMD). The measurements were taken in the midsagittal plane, after bladder emptying, at rest (Figure 1) [47].

Treatment involved Flat Magnetic Stimulation alone or with concomitant local or systemic pharmacological therapy, depending on the patient’s preferences. Flat Magnetic Stimulation was delivered with the DR ARNOLD system (DEKA M.E.L.A. Calenzano, Italy). Treatment was applied for a total of 8 treatments on all patients. Sessions were conducted twice weekly for four consecutive weeks; depending on the patient’s muscular condition, each session lasted 28 min. The overtone/pain protocol (muscle work aimed at muscle inhibition and reduction of pain) was selected after the first two minutes of warm-up for all patients. Any adverse effect was registered and classified according to the Clavien–Dindo classification [A]. The Clavien–Dindo classification is easy to use and has been clinically validated.

One month after the treatment, patients were asked again to score the intensity of pelvic pain using the 10 cm visual analog scale (VAS) and to evaluate their pelvic floor symptoms severity by answering the question, “How much do your pelvic floor symptoms bother you?” on the 5-answer Likert scale. Patients repeated TPU to assess anorectal angle (ARA) and levator ani muscle minimal plane distance (LAMD). The measurements were taken in the midsagittal plane, after bladder emptying, at rest.

The anonymized data were entered into the database by the Authors. Statistical analysis was performed using JMP version 9 software (SAS Institute, Cary, NC, USA). Results were reported as mean ± standard deviation for continuous variables and as number (percentage) for non-continuous variables. Pre- and post-treatment data were compared to obtain objective and subjective results and tested for statistical significance. Differences were tested using a paired *t*-test for continuous data and Fisher’s exact test for non-continuous data. A *p* value < 0.05 was considered statistically significant.

## 3. Results

In total, 11 patients completed baseline evaluation, treatment, and postoperative evaluation in the period of interest. Population characteristics are shown in Table 1. All patients underwent eight sessions of Flat Magnetic Stimulation according to the described protocol. Adjuvant pharmacological treatment was used in five (45.5%) patients. Baseline and post-treatment sonographic findings are reported in Table 2. Specifically, we observed a significant increase in both ARA and LAMD, comparing baseline and post-treatment measurements. Quality of life scale scores at baseline and after treatment are reported in Table 3. A significant improvement in both tools was demonstrated after the treatment. Improvements in quality of life (VAS *p* > 0.001; Likert *p* = 0.001) and sonographic parameters (ARA *p* < 0.001; LAMD *p* = 0.001) remained significant even in patients who received only FMS without adjuvant pharmacological treatment. 

## 4. Discussion

Pelvic floor hypertonicity is a complex disorder that, as previously mentioned, can be caused by multiple triggering events that often coexist with each other. It is, therefore, necessary to evaluate possible causes of traumatic, iatrogenic, postural, and/or antalgic origin. This condition could also be attributed to incorrect pelvic floor activities or poor activities acquired during life, such as continuous voluntary retention of urine or feces [48]. Over the years, some works have appeared in the literature focusing on the diagnosis and treatment of CPPS, increasing the awareness of the complex, multifactorial nature of chronic pelvic pain. So, chronic pelvic pain syndromes could be managed by a multidisciplinary team with the appropriate skills and understanding to address the variety of factors that maintain its condition [1]. Generally, the goals of treatments are reduction of local inflammation, regularization of nerve transmission to decrease pain, and relaxation of contracted muscles. In the case of pelvic floor muscle hypertonic dysfunction, many non-invasive techniques are available. Physiotherapy is the first-line conservative therapy but has the disadvantage of having slow progression and low patient adherence and compliance with treatment [49,50]. The same thing can be said for Kegel exercises, whose effectiveness is reduced because they are often performed inconsistently or incorrectly over time by patients.

**Figure 2:**
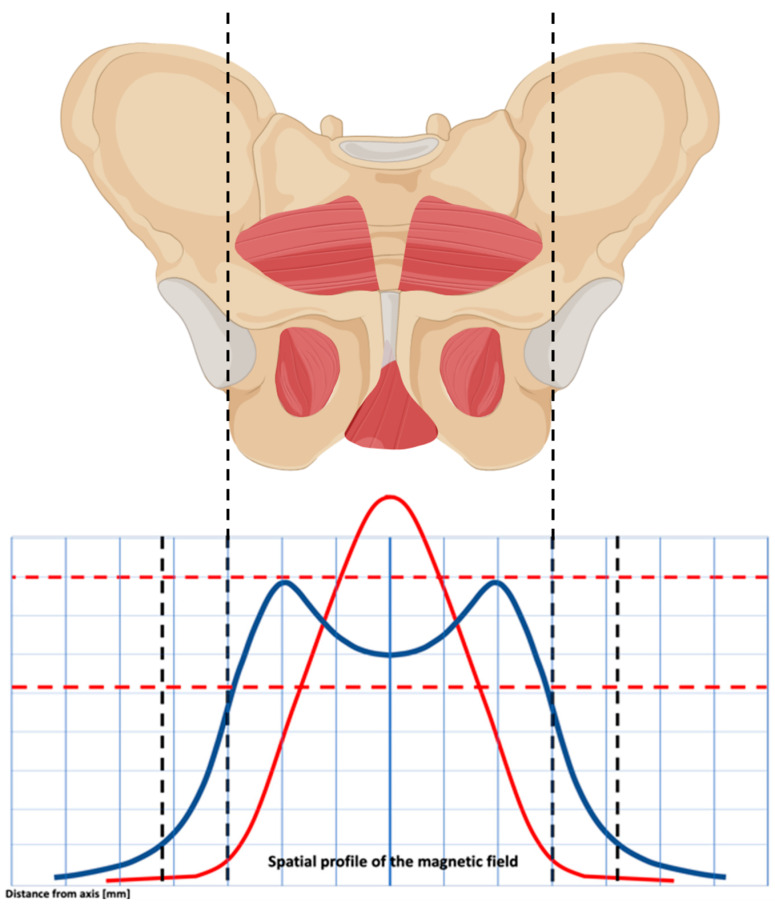
The spatial profile of the magnetic field’s electromagnetic energy results in a double-dome distribution. This allows you to uniformly stimulate the pelvic floor muscles, obtaining a homogeneous effect on the pelvic floor muscles.

According to the evidence in the literature, magnetic stimulation can be considered an alternative method for the treatment of pelvic floor dysfunctions. Since 1998, it has been described as an alternative conservative approach in women with stress and mixed urinary incontinence [43,44,51,52]. Recently published studies have shown that the new TOP Flat Magnetic Stimulation (TOP FMS) technology reduced the symptoms of urge, mixed, and stress incontinence, improving patients’ quality of life without the risk of side effects. [53] (Figure 2).

A fundamental aspect that distinguishes Dr. ARNOLD from other devices is the spatial profile of the electromagnetic stimulation. It is homogeneously distributed up to the top borders, it covers a wider area, and the lateral profiles are better expressed. This conformation allows for a deep, symmetrical, and homogenous distribution of electromagnetic energy, reaching deep neuronal structures inside the pelvis without superficial dispersion. During the procedures, a non-invasive electromagnetic therapeutic device with a main unit and a chair applicator was used. The coil of the chair applicator, which is located in the center of the seat, is intended for therapy of the deep pelvic floor area. The patient is seated on the chair with their perineum in the center of the seat, which helps them feel the stimulation of their pelvic floor and sphincter muscles during stimulation therapy. In order to stimulate the pelvic area, “the chair” can produce an electromagnetic field with a homogenous profile (TOP FMS-TOP Flat Magnetic Stimulation). The beneficial effect of the device in question is due to a greater uniformity of distribution of the magnetic field over a larger area, which allows greater recruitment of muscle fibers without generating different areas in terms of stimulation intensity. Furthermore, the electromagnetic field determines the deep and uniform stimulation of the nerve roots of the sacral nerves (S2–S4) and the pudendal nerve, physiologically responsible for the sensitivity of the perineal region. Electromagnetic energy, stimulation, and deep penetration of the entire pelvic floor are the basis of the effectiveness of this treatment. In the present study, it was shown that relaxing the pelvic floor muscles with the Dr. ARNOLD system is effective. The strengths of this innovative technology are the homogenous profile of stimulation with no differences in intensity between pelvic floor areas, the ergonomic seat, and the opportunity for the patients to stay dressed and not use a vaginal probe. Regarding FMS in PFH, a few studies have reported the use of this device in this peculiar population. Biondo et al. described the use of the device in the treatment of muscle hypertonicity in women with vulvodynia. In these cases, the overtone/pain protocol is based on lower frequencies (around 10 Hz) and low-level electric currents on neuromuscular tissue, bringing to depolarize neurons and PFM decontractions. The homogeneous distribution of the electromagnetic field avoids overstimulation of the already hypersensitive receptors and sensory nerves typical of vulvodynia conditions. In their study, the authors found a significant decrease in PISQ-12 (Pelvic Organ Prolapse/Urinary Incontinence Sexual Questionnaire short form) score and an improvement in vulvodynia symptoms with no side effects [54].

In our study, we aimed to evaluate the effect of Flat Magnetic Stimulation in women with chronic pelvic pain and levator ani hypertonicity on the evolution of symptoms and on the sonographic aspect of the levator ani muscle before and after treatment. After eight sessions of FMS overtone pain protocol, our patients reported an improvement in pain perception and in quality of life, as shown by the significant reduction of VAS and Likert scale scores. Notably, less than half of the patients in our series used adjuvant pharmacological treatment. In addition, we decided to evaluate the effect of magnetic stimulation on the morphological aspect of the levator ani muscle. Through translabial ultrasound, the change of levator ani muscle and anorectal angle in women before and after FMS were collected, and a significant increase in both ARA and LAMD comparing baseline and post-treatment measurements was observed. 

The use of pelvic floor ultrasound to assess pelvic floor muscles is well-established. In fact, it has been shown to be more specific than clinical palpation for measurement of the action of the pelvic floor muscles on the anterior compartment; for these reasons, rehabilitative ultrasound imaging has provided novel access to the structure and behavior of the levator ani muscle and their influence on associated structures [55,56]. It can also provide real-time information as a possible source of biofeedback that can be valuable during re-education of the pelvic floor muscles in patients with pelvic floor dysfunction [57]. In particular, it is well reported that transperineal ultrasound is a feasible and reproducible tool in the assessment of pelvic floor muscle thickness at rest and during contraction as an indirect index of hypertone [42]. On the contrary, there is less data on the relationship between pelvic floor hypertonicity and sonographic muscular measurements. Recent studies have demonstrated how women with chronic pelvic pain were found to have pelvic floor muscle hypertonicity, with a smaller levator hiatal area (LHA) at rest and reduced ability to increase the LHA area on Valsalva maneuver, showing inadequate pelvic floor relaxation [48,58]. With this study, we demonstrated for the first time that magnetic stimulation is able to induce sonographic measurable modifications of pelvic floor muscles consistent with symptom relief. As a consequence, we do think that in the management of pelvic floor hypertonicity, ultrasound imaging can be considered a valid and useful tool to provide important information about the function of the pelvic floor muscles and to monitor the efficacy of the treatment during subsequent observations. 

Strengths of our study include the originality, the prospective design, and the multimodal panel of treatment outcomes evaluation. The most relevant limitation is related to the limited population, which is consistent with the still limited—despite emerging—prevalence of this condition.

## 5. Conclusions

Our study demonstrated that this innovative type of treatment led to a significant improvement in the hypertonicity of the pelvic floor muscles in patients with chronic pelvic pain in terms of symptoms and ultrasound parameters, without discomfort or side effects. Therefore, flat magnetic stimulation represents promising support for the management of chronic pelvic pain related to pelvic floor hypertonicity.

## Figures and Tables

**Table 1:** Population baseline characteristics. Continuous data are reported as mean (SD). Non-continuous data are reported as absolute (relative) frequency.

Age (years)	52.6 ± 12.6
Parity (n)	1.1 ± 1.0
Menopausal status	7 (63.6%)
Chronic pelvic pain	11 (100%)
Obstructed defecation	6 (54.5%)
Dyspareunia	6 (54.5%)
Bladder pain syndrome	5 (45.5%)
Bladder voiding symptoms	4 (36.4%)

**Table 2:** Baseline and post-treatment sonographic findings. ARA: anorectal angle; LAMD: levator ani muscle minimal plane distance.

	Baseline	Post-Treatment	*p*-Value
ARA (°)	84.8 ± 7.7	111.3 ± 5.9	<0.001
LAMD (mm)	39.4 ± 2.9	50.3 ± 3.1	<0.001

**Table 3:** Baseline and post-treatment quality of life findings.

	Baseline	Post-Treatment	*p*-Value
Likert scale	4.4 ± 0.5	1.6 ± 0.7	<0.001
VAS score	75.5 ± 9.3	30.9 ± 19.2	<0.001

## Data Availability

The data presented in this study are available on request from the corresponding author.
